# Twelve Lectures on the Structure of the Central Nervous System

**Published:** 1891-07

**Authors:** 


Twelve Lectures on the Structure of the Central Nervous System. For Physicians and Students. By Dr. Ludwig Edinger, Frankfort-on-the-Main. Second revised edition. Illustrated. Translated by Willis Hall Vittum, M. D., St. Paul, Minn. Edited by C. Eugene Riggs, A. M., M. D., University of Minnesota. Philadelphia (1231 Filbert Street) and London. F. A. Davis, Publisher. 1890. Price, $1.75, net.

This work embodies a course of twelve lectures on the Minute Anatomy of the Central Nervous System of Man, delivered to an audience of practicing physicians.

Its scope is clearly set forth by the author in his preface to the first edition, in which he says that it was his endeavor to lay before his hearers all that had been discovered in regard to the finer structure of the brain. And he modestly adds that it would be absurd to consider it anything more than an introduction to the study.

The second edition embraces the discoveries of four years, and possesses additional value in the chapter devoted to the comparative anatomy and embryology of the brain. It is in this field that the student may hope to gain new light in his studies of the great central organ of man.

To bring such a valuable work within the reach of English-speaking scientists has been the endeavor of Drs. Vittum and Riggs in their American edition. It is a thoroughly able treatise, and a valuable addition to the literature of the subject.

